# 4,4′-Dichloro-*N*,*N*′-(*o*-phenyl­ene)dibenzene­sulfonamide

**DOI:** 10.1107/S1600536808039718

**Published:** 2008-12-20

**Authors:** Julia Krainova, Christopher Dares, A. B. P. Lever

**Affiliations:** a Department of Chemistry, York University, Toronto, Ontario, Canada M3J 1P3

## Abstract

The title compound, C_18_H_14_Cl_2_N_2_O_4_S_2_, is a diamine that is a precursor to a quinonoid bidentate redox-active ligand. The dihedral angles between the central phenyl ring and the end rings are 87.5(1) and 60.7(1)°, while the two end rings make a dihedral angle of 82.5(1)°. The crystal structure is stabilized by two weak inter­molecular N—H⋯O hydrogen bonds, as well as one intra­molecular C—H⋯O and one N—H⋯N hydrogen bond.

## Related literature

For the synthesis of related substituted *o*-phenyl­enediamines, see: Massacret *et al.* (1999 ▶). For background to the use of substituted *o*-benzoquinones as ligands, see: Masui & Lever (1993 ▶); Kalinina *et al.* (2008 ▶) and references therein.
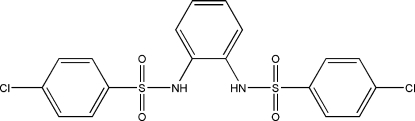


## Experimental

### 

#### Crystal data

C_18_H_14_Cl_2_N_2_O_4_S_2_
*M* *_r_* = 457.33
Triclinic, 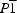

*a* = 7.7225 (4) Å
*b* = 11.1920 (4) Å
*c* = 11.9325 (5) Å
α = 109.669 (2)°
β = 91.420 (2)°
γ = 101.782 (2)°
*V* = 945.79 (7) Å^3^
*Z* = 2
Mo *K*α radiation
μ = 0.59 mm^−1^
*T* = 150 (1) K
0.40 × 0.36 × 0.30 mm

#### Data collection

Bruker–Nonius KappaCCD diffractometer
Absorption correction: multi-scan (*SORTAV*; Blessing, 1995 ▶) *T*_min_ = 0.748, *T*_max_ = 0.876
8650 measured reflections
4235 independent reflections
3386 reflections with *I* > 2σ(*I*)
*R*_int_ = 0.032

#### Refinement

*R*[*F* ^2^ > 2σ(*F* ^2^)] = 0.046
*wR*(*F* ^2^) = 0.122
*S* = 1.08
4235 reflections
261 parameters
H atoms treated by a mixture of independent and constrained refinement
Δρ_max_ = 0.48 e Å^−3^
Δρ_min_ = −0.69 e Å^−3^

### 

Data collection: *COLLECT* (Nonius, 2002 ▶); cell refinement: *DENZO-SMN* (Otwinowski & Minor, 1997 ▶); data reduction: *DENZO-SMN*; program(s) used to solve structure: *SHELXS97* (Sheldrick, 2008 ▶); program(s) used to refine structure: *SHELXL97* (Sheldrick, 2008 ▶); molecular graphics: *ORTEP-3 for Windows* (Farrugia, 1997 ▶); software used to prepare material for publication: *WinGX* (Farrugia, 1999 ▶).

## Supplementary Material

Crystal structure: contains datablocks global, I. DOI: 10.1107/S1600536808039718/bx2184sup1.cif

Structure factors: contains datablocks I. DOI: 10.1107/S1600536808039718/bx2184Isup2.hkl

Additional supplementary materials:  crystallographic information; 3D view; checkCIF report

## Figures and Tables

**Table 1:** Hydrogen-bond geometry (Å, °)

*D*—H⋯*A*	*D*—H	H⋯*A*	*D*⋯*A*	*D*—H⋯*A*
N1—H1⋯O4^i^	0.87 (3)	2.12 (3)	2.936 (3)	157 (2)
N2—H2⋯O2^ii^	0.85 (3)	2.30 (3)	3.107 (3)	159 (2)
N1—H1⋯N2	0.87 (3)	2.45 (3)	2.811 (3)	106 (2)
C6—H6⋯O1	0.95	2.22	2.900 (3)	128

Symmetry codes: (i) 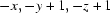; (ii) 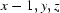.

**Fig. 1.**
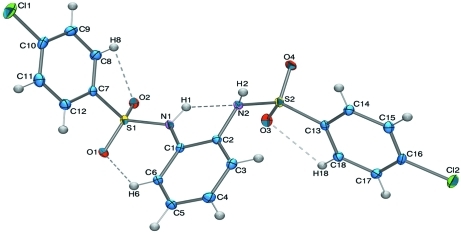
A view of (I) showing the molecular structure and intramolecular H bonding present. Displacement ellipsoids are drawn at the 30% probability level.

**Fig. 2.**
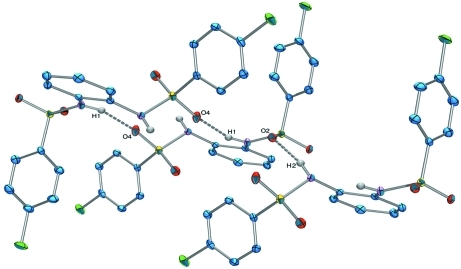
Crystal packing diagram of (I) showing the intermolecular H bonds present. H atoms not involved in H bonding are not shown. Displacement ellipsoids are drawn at the 30% probability level.

## supplementary crystallographic information

### Comment

Benzoquinonediimine compounds have been extensively studied as ligands in metal complex systems (Kalinina *et al.*, 2008). As free ligands, they exist in the diamine form, however, they have the ability to bind to a metal in one of three oxidation states: as *o*-phenylenediamine, its one-electron oxidized *o*-semiquinonediiminate, or its doubly oxidized and strongly π-accepting *o*-benzoquinonediimine form. In addition to being redox-active, these ligands also often exhibit non-innocent behaviour. Ruthenium complexes of *o*-benzoquinonediimines possess highly covalent bonds. The extent of electronic coupling between the metal and diimine ligand can be tuned by using substituented *o*-benzoquinones (Masui & Lever, 1993, and Kalinina *et al.*, 2008). We present here the synthesis and crystal structure of the title compound (I). In (I) (Fig. 1), highly electron-withdrawing groups (*p*-chlorophenylsulfonyl, *PCPS*) are bound to the amine N atoms, and are expected to exhibit a greater covalent metal-benzoquinonediimine ligand bond character than the unsubstituted diimine. The C1—N1—S1—C7 dihedral angle is 81.0 (2) °, while the C2—N2—S2—C13 dihedral angle is only 68.6 (2)°. The p-chlorophenyl rings are essentially perpendicular to the N—S bond, likely due to steric hindrance and intermolecular H bonding between the *ortho* H atoms, and the sulfonyl O atoms (Fig. 1). The crystal structure is stabilized by two weak intermolecular N—H···O hydrogen bonds (Fig. 2), as well as three intramolecular C—H···O and one N—H···N hydrogen bonds which increases the stability of the crystal, (Table 1). The bonds parameters are similar to those in the other arylsulfonamides.

### Experimental

(I) was synthesized for the first time according to methods described by Massacret *et al.*, 1999, using *p*-chlorophenylsulfonyl chloride (10 mmol) as the arenesulfonyl chloride. *o*-phenylenediamine (540 mg, 5 mmol) was twice sublimed under reduced pressure prior to use. After purification, (I) was dissolved in a minimal amount of ethanol, and then added to an aqueous solution of CuCl_2_ (20 ml, 0.15 *M*). Colorless block-like crystals suitable for *x*-ray diffraction studies were obtained after allowing the solution to stand for 2 weeks.

### Refinement

All H atoms attached to C atoms were added in calculated locations and constrained to ride on their parent atoms with *U*_iso_(H) = 1.2*U*_eq_(C). Both H atoms attached to N atoms were located in the electron-density difference map, with *U*_iso_(H) = 1.2*U*_eq_(N).

### Crystal data

**Table:**

C_18_H_14_Cl_2_N_2_O_4_S_2_	*Z* = 2
*M**_r_* = 457.33	*F*(000) = 468
Triclinic, *P*1	*D*_x_ = 1.606 Mg m^−^^3^
Hall symbol: -P 1	Mo *K*α radiation, λ = 0.71073 Å
*a* = 7.7225 (4) Å	Cell parameters from 4103 reflections
*b* = 11.1920 (4) Å	θ = 2.6–27.5°
*c* = 11.9325 (5) Å	µ = 0.59 mm^−^^1^
α = 109.669 (2)°	*T* = 150 K
β = 91.420 (2)°	Prism, colourless
γ = 101.782 (2)°	0.40 × 0.36 × 0.30 mm
*V* = 945.79 (7) Å^3^	

### Data collection

**Table:**

Bruker–Nonius KappaCCD diffractometer	4235 independent reflections
Radiation source: fine-focus sealed tube	3386 reflections with *I* > 2σ(*I*)
graphite	*R*_int_ = 0.032
φ scans and ω scans with κ offsets	θ_max_ = 27.5°, θ_min_ = 2.7°
Absorption correction: multi-scan (*SORTAV*; Blessing, 1995)	*h* = −10→9
*T*_min_ = 0.748, *T*_max_ = 0.876	*k* = −14→14
8650 measured reflections	*l* = −14→15

### Refinement

**Table:**

Refinement on *F*^2^	Primary atom site location: structure-invariant direct methods
Least-squares matrix: full	Secondary atom site location: difference Fourier map
*R*[*F*^2^ > 2σ(*F*^2^)] = 0.046	Hydrogen site location: inferred from neighbouring sites
*wR*(*F*^2^) = 0.122	H atoms treated by a mixture of independent and constrained refinement
*S* = 1.08	*w* = 1/[σ^2^(*F*_o_^2^) + (0.06*P*)^2^ + 0.6434*P*] where *P* = (*F*_o_^2^ + 2*F*_c_^2^)/3
4235 reflections	(Δ/σ)_max_ < 0.001
261 parameters	Δρ_max_ = 0.48 e Å^−^^3^
0 restraints	Δρ_min_ = −0.69 e Å^−^^3^

### Special details

**Table:**

Geometry. All s.u.'s (except the s.u. in the dihedral angle between two l.s. planes) are estimated using the full covariance matrix. The cell s.u.'s are taken into account individually in the estimation of s.u.'s in distances, angles and torsion angles; correlations between s.u.'s in cell parameters are only used when they are defined by crystal symmetry. An approximate (isotropic) treatment of cell s.u.'s is used for estimating s.u.'s involving l.s. planes.
Refinement. Refinement of *F*^2^ against ALL reflections. The weighted *R*-factor *wR* and goodness of fit *S* are based on *F*^2^, conventional *R*-factors *R* are based on *F*, with *F* set to zero for negative *F*^2^. The threshold expression of *F*^2^ > 2σ(*F*^2^) is used only for calculating *R*-factors(gt) *etc*. and is not relevant to the choice of reflections for refinement. *R*-factors based on *F*^2^ are statistically about twice as large as those based on *F*, and *R*- factors based on ALL data will be even larger.

### Fractional atomic coordinates and isotropic or equivalent isotropic displacement parameters (Å^2^)

**Table:**

	*x*	*y*	*z*	*U*_iso_*/*U*_eq_	
C1	0.1732 (3)	0.8391 (2)	0.77764 (19)	0.0171 (4)	
C2	−0.0029 (3)	0.8152 (2)	0.72753 (19)	0.0171 (4)	
C3	−0.1000 (3)	0.9105 (2)	0.7632 (2)	0.0222 (5)	
H3	−0.2178	0.8935	0.7273	0.027*	
C4	−0.0274 (3)	1.0303 (2)	0.8506 (2)	0.0262 (5)	
H4	−0.0940	1.0957	0.8744	0.031*	
C5	0.1440 (3)	1.0528 (2)	0.9026 (2)	0.0257 (5)	
H5	0.1934	1.1333	0.9646	0.031*	
C6	0.2444 (3)	0.9599 (2)	0.8657 (2)	0.0225 (5)	
H6	0.3630	0.9785	0.9008	0.027*	
C7	0.3601 (3)	0.6325 (2)	0.8929 (2)	0.0195 (5)	
C8	0.3196 (3)	0.4973 (2)	0.8469 (2)	0.0270 (5)	
H8	0.3392	0.4524	0.7669	0.032*	
C9	0.2500 (4)	0.4285 (3)	0.9191 (2)	0.0314 (6)	
H9	0.2220	0.3360	0.8891	0.038*	
C10	0.2220 (3)	0.4956 (3)	1.0349 (2)	0.0284 (6)	
C11	0.2637 (4)	0.6307 (3)	1.0819 (2)	0.0310 (6)	
H11	0.2448	0.6752	1.1622	0.037*	
C12	0.3334 (3)	0.7000 (2)	1.0100 (2)	0.0266 (5)	
H12	0.3625	0.7925	1.0405	0.032*	
C13	−0.2243 (3)	0.7466 (2)	0.45683 (19)	0.0189 (4)	
C14	−0.4080 (3)	0.7072 (2)	0.4534 (2)	0.0210 (5)	
H14	−0.4581	0.6378	0.4801	0.025*	
C15	−0.5169 (3)	0.7697 (2)	0.4109 (2)	0.0225 (5)	
H15	−0.6425	0.7440	0.4078	0.027*	
C16	−0.4392 (3)	0.8706 (2)	0.3730 (2)	0.0236 (5)	
C17	−0.2571 (3)	0.9119 (2)	0.3774 (2)	0.0264 (5)	
H17	−0.2077	0.9826	0.3523	0.032*	
C18	−0.1476 (3)	0.8486 (2)	0.4190 (2)	0.0233 (5)	
H18	−0.0221	0.8745	0.4217	0.028*	
N1	0.2723 (3)	0.7422 (2)	0.73182 (17)	0.0203 (4)	
H1	0.222 (4)	0.673 (3)	0.672 (3)	0.024*	
N2	−0.0870 (3)	0.68766 (18)	0.64404 (17)	0.0191 (4)	
H2	−0.188 (4)	0.654 (3)	0.660 (2)	0.023*	
O1	0.5452 (2)	0.84388 (16)	0.87507 (14)	0.0235 (4)	
O2	0.5228 (2)	0.63638 (16)	0.70620 (14)	0.0232 (4)	
O3	0.0908 (2)	0.70716 (18)	0.48023 (15)	0.0289 (4)	
O4	−0.1729 (2)	0.52320 (16)	0.44441 (16)	0.0311 (4)	
S1	0.44267 (7)	0.71968 (5)	0.79949 (5)	0.01842 (15)	
S2	−0.08688 (7)	0.65782 (5)	0.50003 (5)	0.02006 (15)	
Cl1	0.13235 (9)	0.40953 (8)	1.12463 (7)	0.0420 (2)	
Cl2	−0.57712 (9)	0.94836 (6)	0.31859 (6)	0.03448 (18)	

### Atomic displacement parameters (Å^2^)

**Table:**

	*U*^11^	*U*^22^	*U*^33^	*U*^12^	*U*^13^	*U*^23^
C1	0.0178 (11)	0.0206 (11)	0.0154 (10)	0.0057 (9)	0.0010 (8)	0.0087 (9)
C2	0.0169 (11)	0.0195 (11)	0.0155 (10)	0.0034 (9)	0.0013 (8)	0.0072 (9)
C3	0.0196 (11)	0.0263 (12)	0.0237 (12)	0.0074 (10)	0.0014 (9)	0.0111 (10)
C4	0.0310 (13)	0.0252 (12)	0.0241 (12)	0.0123 (11)	0.0056 (10)	0.0073 (10)
C5	0.0331 (14)	0.0221 (12)	0.0188 (11)	0.0067 (10)	−0.0031 (10)	0.0032 (10)
C6	0.0243 (12)	0.0210 (11)	0.0196 (11)	0.0035 (9)	−0.0048 (9)	0.0051 (9)
C7	0.0163 (11)	0.0256 (12)	0.0180 (11)	0.0072 (9)	−0.0012 (8)	0.0080 (9)
C8	0.0342 (14)	0.0255 (12)	0.0223 (12)	0.0102 (11)	0.0037 (10)	0.0075 (10)
C9	0.0379 (15)	0.0261 (13)	0.0343 (14)	0.0084 (11)	0.0036 (12)	0.0152 (12)
C10	0.0251 (13)	0.0389 (15)	0.0296 (13)	0.0096 (11)	0.0007 (10)	0.0216 (12)
C11	0.0324 (14)	0.0425 (15)	0.0190 (12)	0.0094 (12)	0.0044 (10)	0.0111 (11)
C12	0.0285 (13)	0.0278 (13)	0.0221 (12)	0.0066 (11)	0.0007 (10)	0.0070 (10)
C13	0.0195 (11)	0.0224 (11)	0.0141 (10)	0.0064 (9)	−0.0012 (8)	0.0046 (9)
C14	0.0193 (11)	0.0251 (12)	0.0205 (11)	0.0063 (9)	0.0027 (9)	0.0096 (9)
C15	0.0181 (11)	0.0296 (13)	0.0212 (11)	0.0089 (10)	0.0029 (9)	0.0086 (10)
C16	0.0312 (13)	0.0268 (12)	0.0152 (11)	0.0162 (11)	−0.0017 (9)	0.0052 (9)
C17	0.0319 (14)	0.0242 (12)	0.0243 (12)	0.0032 (10)	−0.0019 (10)	0.0120 (10)
C18	0.0199 (12)	0.0268 (12)	0.0226 (12)	0.0023 (10)	−0.0006 (9)	0.0096 (10)
N1	0.0183 (10)	0.0222 (10)	0.0185 (9)	0.0057 (8)	−0.0048 (8)	0.0046 (8)
N2	0.0154 (9)	0.0202 (10)	0.0208 (10)	0.0005 (8)	−0.0030 (8)	0.0084 (8)
O1	0.0170 (8)	0.0242 (9)	0.0245 (8)	−0.0009 (7)	−0.0062 (7)	0.0062 (7)
O2	0.0180 (8)	0.0303 (9)	0.0220 (8)	0.0080 (7)	0.0014 (6)	0.0087 (7)
O3	0.0194 (9)	0.0431 (11)	0.0265 (9)	0.0115 (8)	0.0040 (7)	0.0126 (8)
O4	0.0343 (10)	0.0182 (8)	0.0327 (10)	0.0087 (8)	−0.0144 (8)	−0.0017 (7)
S1	0.0146 (3)	0.0234 (3)	0.0175 (3)	0.0048 (2)	−0.0017 (2)	0.0074 (2)
S2	0.0180 (3)	0.0221 (3)	0.0189 (3)	0.0076 (2)	−0.0027 (2)	0.0043 (2)
Cl1	0.0365 (4)	0.0621 (5)	0.0440 (4)	0.0103 (3)	0.0061 (3)	0.0403 (4)
Cl2	0.0453 (4)	0.0365 (4)	0.0275 (3)	0.0237 (3)	−0.0036 (3)	0.0107 (3)

### Geometric parameters (Å, °)

**Table:**

C1—C6	1.395 (3)	C11—H11	0.9500
C1—C2	1.408 (3)	C12—H12	0.9500
C1—N1	1.422 (3)	C13—C14	1.392 (3)
C2—C3	1.385 (3)	C13—C18	1.393 (3)
C2—N2	1.443 (3)	C13—S2	1.769 (2)
C3—C4	1.387 (3)	C14—C15	1.382 (3)
C3—H3	0.9500	C14—H14	0.9500
C4—C5	1.385 (4)	C15—C16	1.386 (3)
C4—H4	0.9500	C15—H15	0.9500
C5—C6	1.383 (3)	C16—C17	1.381 (4)
C5—H5	0.9500	C16—Cl2	1.743 (2)
C6—H6	0.9500	C17—C18	1.387 (3)
C7—C8	1.388 (3)	C17—H17	0.9500
C7—C12	1.390 (3)	C18—H18	0.9500
C7—S1	1.764 (2)	N1—S1	1.6329 (18)
C8—C9	1.387 (3)	N1—H1	0.87 (3)
C8—H8	0.9500	N2—S2	1.637 (2)
C9—C10	1.380 (4)	N2—H2	0.85 (3)
C9—H9	0.9500	O1—S1	1.4296 (17)
C10—C11	1.388 (4)	O2—S1	1.4359 (17)
C10—Cl1	1.735 (3)	O3—S2	1.4278 (18)
C11—C12	1.388 (4)	O4—S2	1.4312 (17)
			
C6—C1—C2	118.0 (2)	C14—C13—C18	121.3 (2)
C6—C1—N1	123.3 (2)	C14—C13—S2	119.09 (17)
C2—C1—N1	118.56 (19)	C18—C13—S2	119.43 (18)
C3—C2—C1	120.5 (2)	C15—C14—C13	119.5 (2)
C3—C2—N2	119.54 (19)	C15—C14—H14	120.3
C1—C2—N2	119.79 (19)	C13—C14—H14	120.3
C2—C3—C4	120.8 (2)	C14—C15—C16	118.7 (2)
C2—C3—H3	119.6	C14—C15—H15	120.6
C4—C3—H3	119.6	C16—C15—H15	120.6
C5—C4—C3	118.7 (2)	C17—C16—C15	122.4 (2)
C5—C4—H4	120.6	C17—C16—Cl2	119.05 (19)
C3—C4—H4	120.6	C15—C16—Cl2	118.53 (19)
C6—C5—C4	121.1 (2)	C16—C17—C18	119.0 (2)
C6—C5—H5	119.4	C16—C17—H17	120.5
C4—C5—H5	119.4	C18—C17—H17	120.5
C5—C6—C1	120.7 (2)	C17—C18—C13	119.1 (2)
C5—C6—H6	119.6	C17—C18—H18	120.5
C1—C6—H6	119.6	C13—C18—H18	120.5
C8—C7—C12	121.3 (2)	C1—N1—S1	127.72 (16)
C8—C7—S1	119.10 (18)	C1—N1—H1	117.6 (18)
C12—C7—S1	119.63 (18)	S1—N1—H1	112.0 (18)
C9—C8—C7	119.2 (2)	C2—N2—S2	119.94 (15)
C9—C8—H8	120.4	C2—N2—H2	115.3 (19)
C7—C8—H8	120.4	S2—N2—H2	110.4 (18)
C10—C9—C8	119.5 (2)	O1—S1—O2	119.90 (10)
C10—C9—H9	120.3	O1—S1—N1	108.49 (10)
C8—C9—H9	120.3	O2—S1—N1	105.21 (10)
C9—C10—C11	121.6 (2)	O1—S1—C7	107.30 (10)
C9—C10—Cl1	119.4 (2)	O2—S1—C7	107.80 (11)
C11—C10—Cl1	119.0 (2)	N1—S1—C7	107.61 (10)
C12—C11—C10	119.1 (2)	O3—S2—O4	121.51 (12)
C12—C11—H11	120.4	O3—S2—N2	106.60 (10)
C10—C11—H11	120.4	O4—S2—N2	105.45 (11)
C11—C12—C7	119.3 (2)	O3—S2—C13	107.47 (11)
C11—C12—H12	120.3	O4—S2—C13	106.22 (10)
C7—C12—H12	120.3	N2—S2—C13	109.20 (10)

### Hydrogen-bond geometry (Å, °)

**Table:**

*D*—H···*A*	*D*—H	H···*A*	*D*···*A*	*D*—H···*A*
N1—H1···O4^i^	0.87 (3)	2.12 (3)	2.936 (3)	157 (2)
N2—H2···O2^ii^	0.85 (3)	2.30 (3)	3.107 (3)	159 (2)
N1—H1···N2	0.87 (3)	2.45 (3)	2.811 (3)	106 (2)
C6—H6···O1	0.95	2.22	2.900 (3)	128
C8—H8···O2	0.95	2.58	2.931 (3)	103
C18—H18···O3	0.95	2.50	2.887 (3)	104

Symmetry codes: (i) −*x*, −*y*+1, −*z*+1; (ii) *x*−1, *y*, *z*.
